# Few-shot RUL prediction for engines based on CNN-GRU model

**DOI:** 10.1038/s41598-024-66377-3

**Published:** 2024-07-11

**Authors:** Shuhan Sun, Jiongqi Wang, Yaqi Xiao, Jian Peng, Xuanying Zhou

**Affiliations:** 1 School of Science, National University of Defense Technology, Changsha, 410073 China; 2 School of Design, Hunan University, Changsha, 410073 China

**Keywords:** Few shot, CNN, GRU, Features extraction, RUL prediction, Applied mathematics, Aerospace engineering, Computer science

## Abstract

In the realm of prognosticating the remaining useful life (RUL) of pivotal components, such as aircraft engines, a prevalent challenge persists where the available historical life data often proves insufficient. This insufficiency engenders obstacles such as impediments in performance degradation feature extraction, inadequacies in capturing temporal relationships comprehensively, and diminished predictive accuracy. To address this issue, a 1D CNN-GRU prediction model for few-shot conditions is proposed in this paper. In pursuit of more comprehensive data feature extraction and enhanced RUL prognostication precision, the Convolutional Neural Network (CNN) is selected for its capacity to discern high-dimensional features amid the intricate dynamics of the data. Concurrently, the Gated Recurrent Unit (GRU) network is leveraged for its robust capability in extracting temporal features inherent within the data. We combine the two to construct a CNN-GRU hybrid network. Moreover, the integration of data distribution alongside correlation and monotonicity indices is employed to winnow the input of multi-sensor monitoring parameters into the CNN-GRU network. Finally, the engine RULs are predicted by the trained model. In this paper, experiments are conducted on a sub-dataset of the National Aeronautics and Space Administration (NASA) C-MAPSS multi-constraint dataset to validate the effectiveness of the method. Experimental results have demonstrated that this method has high accuracy in RUL prediction tasks, which can powerfully demonstrate its effectiveness.

## Introduction

Predictive and Health Management (PHM) is an effective methodology that utilizes data analytics, fault diagnosis, and prediction techniques to assess the safety and reliability and predict the state of complex systems under actual operating conditions. Remaining Useful Life (RUL), which refers to the prediction of future system failure events based on currently available monitoring information, is the core of PHM. This technique aims to accurately predict the remaining operating time of a system before it fails, which provides a key basis for formulating a scientific and reasonable maintenance strategy. In the flight safety and security system, aircraft engines are the key to ensuring the safety of air transportation^1^. The scientific and reliable prediction of RUL can provide decision-makers with valuable reference information for implementing appropriate maintenance measures, thereby effectively mitigating the severe consequences of unplanned engine shutdowns and ensuring flight safety.

The methods for predicting the lifespan of systems can be broadly categorized into two groups: physics-based modeling methods and data-driven methods. The successful application of physics-based modeling methods relies on obtaining accurate and reliable physics models. However, with contemporary equipment becoming increasingly complex, nonlinear, and high-dimensional, accurately modeling the evolutionary laws of their health states using physics-based methods often proves challenging or cost-prohibitive in terms of acquiring failure mechanism models. Consequently, to some extent, the widespread application of this type of method is limited. On the other hand, data-driven lifetime prediction methods do not necessitate the establishment of intricate system physics models. In the era of big data, developing deep learning-based data-driven remaining lifetime prediction methods has emerged as a current mainstream approach and research focus^2^. The application of neural network-based methods has witnessed extensive development and utilization in learning the mapping from collected feature data to relevant remaining useful life (RUL). A notable advantage of neural networks lies in their capability to effectively model highly nonlinear, complex, and multi-dimensional systems without relying on prior physical knowledge^1^. The model can directly utilize various types of system data, such as multi-source raw sensor monitoring data, as input. Huang et al.^3^ used traditional multi-layer perceptron (MLP) methods to predict the remaining useful life of bearings and confirmed that the modeling method was superior to reliability-based methods. Tian^4^ established an artificial neural network (ANN) model for estimating the RUL of equipment. The model takes as input the age and multiple state monitoring measurement values at current and previous inspection points and outputs the percentage of equipment life. Fink et al.^5^ proposed a multi-valued neural network method for the reliable and degradation time series prediction problem using a method with multi-valued neurons in a multi-layer neural network. The method was experimentally verified by a case study of railway switch system degradation. Malhi et al.^6^ proposed a recursive neural network (RNN) method based on competitive learning for the long-term prediction of machine health status. To overcome the disadvantage that the usual neural network-based methods cannot directly obtain the RUL estimation confidence limit, Khawaja et al.^7^ introduced a confidence prediction neural network method with a confidence distribution node. However, the data-driven lifetime prediction methods require sufficient and complete monitoring data; however, as industrial manufacturing levels develop, high-reliability equipment (such as aerospace vehicles) has few-shot problems such as insufficient samples, high cost, and long cycle for obtaining lifetime information, sensor failure causing data loss^8,9^; The reliability of life prediction results based on such data cannot be guaranteed, thereby presenting numerous challenges and complexities in the application of big data theory to life prediction methods.

The crux of data-driven remaining useful life (RUL) prediction lies in capturing the degradation characteristics inherent in time series data. Therefore, the accuracy of RUL prediction is contingent upon whether the constructed model has effectively assimilated temporal information^10^. Conventional model-based residual lifetime prediction methods typically rely on expert knowledge and prior information, necessitating manual feature extraction and selection. This process becomes more challenging and time-consuming for few-shot problems^6,11–13^. Currently, the existing life prediction methods based on deep learning primarily focus on individual time series networks, thereby limiting their ability to effectively extract and utilize profound information across data. Convolutional Neural Network (CNN) are a class of deep feedforward neural networks that possess convolutional computation capabilities, enabling them to achieve multiple nonlinear transformations through a deep convolutional framework to capture the intricate dynamic features of time-series data in high-dimensional space. Specifically, alternating stacks of convolutional layers and pooling layers are employed to extract abstract spatial features, while backpropagation is utilized for determining the parameters of the convolutional kernel, ultimately resulting in the extraction of final features after traversing multiple hidden layers. In few-shot datasets, the utilization of excessively complex models poses a higher risk of overfitting. CNN mitigates this concern by reducing model parameters and enhancing generalization and stability through shared convolutional kernel mechanisms. Moreover, the convolutional layers generate feature maps that are subsequently processed in the pooling layer to extract essential local information. This process effectively reduces feature dimensionality and model parameters, thereby mitigating overfitting risks in few-shot conditions. Dropout is a technique that can help reduce data overfitting during the training of neural networks, especially when dealing with the few-shot training dataset^15^. It can prevent complex co-adaptation to the training data and avoid repeatedly extracting the same features. However, CNN also has some limitations in feature extraction. Although CNN is very powerful in capturing local spatial feature information, in the problem of long-term prediction for high-reliability equipment with temporal data that involves more complex temporal correlation, CNN may be limited in handling such sequential information.

To address the issue of time series data modeling, Recurrent Neural Network (RNN) introduced the concept of hidden state, enabling feature extraction from sequential data and subsequent conversion to output. The inherent sequence and list-related nature of RNN is revealed through their chaining feature, making them the most suitable neural network architectures for this type of data. However, a limitation of RNN lies in their short-term memory capacity as they struggle to effectively transfer information from earlier time steps to later ones when dealing with sufficiently long sequences. This is attributed to the challenge of gradient vanishing and explosion faced by RNN during backpropagation. To address the limitations of RNN in learning long-term dependencies, particularly in time series prediction problems involving extended periods, Hochreiter and Schmidhuber^16^ introduced Long Short-Term Memory (LSTM) in 1997. However, due to its intricate network structure, LSTM exhibits suboptimal accuracy and relatively lower prediction efficiency and speed. In 2014, Cho et al.^17^ proposed a variant known as Gate Recurrent Unit (GRU), which features a simpler internal architecture with fewer parameters compared to LSTM. Consequently, GRU models may offer faster training speeds in few-shot datasets while being less prone to overfitting. Due to its relatively simple gate mechanism, the gradient of GRU can be easily propagated during backpropagation, leading to enhanced stability in handling long-term dependencies, particularly in scenarios with few-shot datasets and limited sample sizes. Moreover, GRU not only effectively captures temporal correlations among data but also enhances its capacity to approximate non-linear patterns. However, it fails to reveal the internal patterns within each dimension of the data.

The aforementioned literature suggests that data-driven methods for predicting remaining useful life (RUL) have shown some progress, but there is still room for improvement in terms of prediction accuracy when dealing with few-shot problems. Achieving precise and efficient RUL prediction solely through the use of a single method poses challenges. However, by leveraging the strengths of different networks and adopting a sequential approach to predict RUL, it is possible to enhance model accuracy. In recent years, extensive and in-depth research has been conducted by several scholars on the application of deep mixture models to prediction problems. In 2021, Zhou et al.^18^ proposed a non-intrusive load decomposition method based on a hybrid deep learning model that combines CNN and LSTM. The aim was to enhance the performance of monitoring and decomposing electrical equipment energy consumption load. This method achieved a test accuracy of 98% on the UK-DALE dataset.Building upon this work, Marei et al.^19^ introduced a hybrid CNN-LSTM model with an embedded transfer learning mechanism in 2022 for predicting the Remaining Useful Life (RUL) of a tool. The innovation lies in incorporating transfer learning to reduce the required amount of training data sets for the deep learning model while improving prediction accuracy through the design of a CNN-LSTM hybrid model. Abhijeet et al.^20^, on the other hand, proposed a hybrid deep learning model that combines Convolutional Neural Network (CNN) and Bi-directional Gated Recurrent Unit (Bi-GRU) for predicting sunspot numbers (SSN). They also employed gradient-residual-correction (GRC) technique to enhance prediction accuracy.Furthermore, Md. Rayhan et al.^21^ presented an ensemble method that leverages efficient feature extraction from Convolutional Neural Network (CNN), Long Short-Term Memory (LSTM), and Gated Recurrent Unit (GRU). Their approach addresses hidden local speech feature extraction issues encountered in most studies but overlooks global long-term context representation challenges associated with speech signals. Overall, these studies contribute significantly to advancing predictive modeling using deep mixture models across various domains such as energy consumption monitoring, tool RUL prediction, sunspot number forecasting, and speech signal analysis. Therefore, this paper aims to integrate the advantages of CNN and GRU in data mining and propose a joint CNN-GRU model-based method for RUL prediction under few-shot conditions, which will be applied to aviation engine prognostics. The proposed method involves pre-screening the multi-source sensor detection data and utilizing CNN to extract temporal degradation features from the original data, thereby forming a high-dimensional predictive feature vector. To address the challenges of complex structure and low prediction accuracy associated with few-shot conditions in LSTM networks, we employ a GRU network that enables accurate prediction of RUL. In the experimental part, to simulate the few-shot conditions, the FD001 sub-dataset of the multi-scenario C-MAPSS dataset provided by NASA was selected for the experiment. The prediction results of the proposed model and other related models were compared through experiments to verify the accuracy of the proposed prediction model. The organization of this paper is as follows: In “Proposed deep learning architecture”, the basic principles of the proposed method are briefly introduced; in “Proposed method”, the deep learning network structure and remaining useful life prediction model under the few-shot conditions are proposed; in “Experiments and results”, the proposed method was experimentally verified using the C-MAPSS dataset; finally, in “Discussion”, the conclusion is drawn.

## Proposed deep learning architecture

### Convolutional neural network

**Figure 1:**
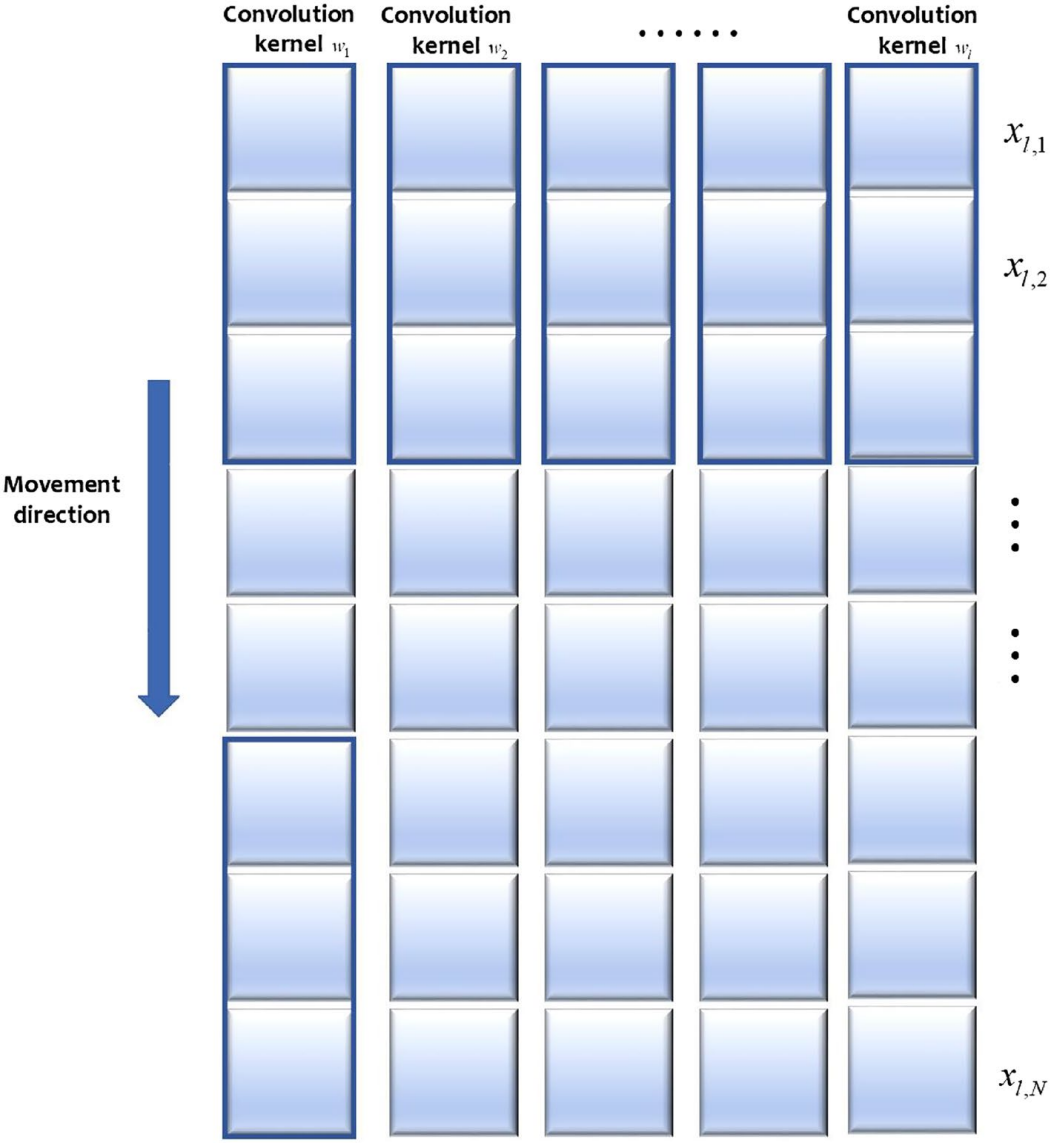
Sliding direction of 1D CNN convolution kernel.

LeCun proposed the Convolutional Neural Network (CNN) in 1988, which is mainly used in the field of image processing. The uniqueness of CNN lies in the introduction of concepts such as local receptive fields, shared spatial weights, and spatial pooling. Today, it has achieved great success in many research and industrial fields, including computer vision, natural language processing, speech recognition, etc. Its structure mainly includes the input layer, convolutional layer, pooling layer, fully connected layer, and output layer. Among them, the convolutional layer and pooling layer are the core components of CNN. Firstly, the convolution kernel traverses through the entire input time series data to generate a higher-level and more abstract feature space. Then, the pooling layer compresses each generated degraded feature for secondary feature extraction and dimensionality reduction by selecting important features at a higher level. Finally, the feature matrix outputted from the convolutional layers is scanned through traversal to produce new sequence features as inputs for subsequent convolutional layers. Traditional CNN is typically two-dimensional, designed for image data where the convolution kernel slides over the width and height of the input image to perform convolutions. However, traditional CNN is not applicable for analyzing sequence data with one-dimensional features, such as time series or text data, as these tasks necessitate considering correlations solely along a specific dimension of the data. Consequently, 1D Convolutional Neural Network (1D CNN) has emerged and gained widespread usage. The key difference between 1D CNN and 2D CNN is the dimension of the input data processed and the moving dimension of the convolution kernel. In the case of time series data, the convolution kernel of a 1D CNN is solely convolved along the temporal sequence of steps, as shown in Fig. 1.

Considering that the engine features utilized in this study for remaining useful life prediction are obtained from diverse sensors, the spatial relationship between adjacent features within a data sample is not highly significant. Moreover, the sequential order of these features does not accurately represent their actual correlation or spatial relationship; rather, it merely reflects their arrangement within the dataset. Consequently, in our application, despite having two-dimensional input and corresponding features, we only need to consider the temporal dimension when analyzing data relationships. Hence, an one-dimensional convolutional filter is employed.

**Figure 2:**
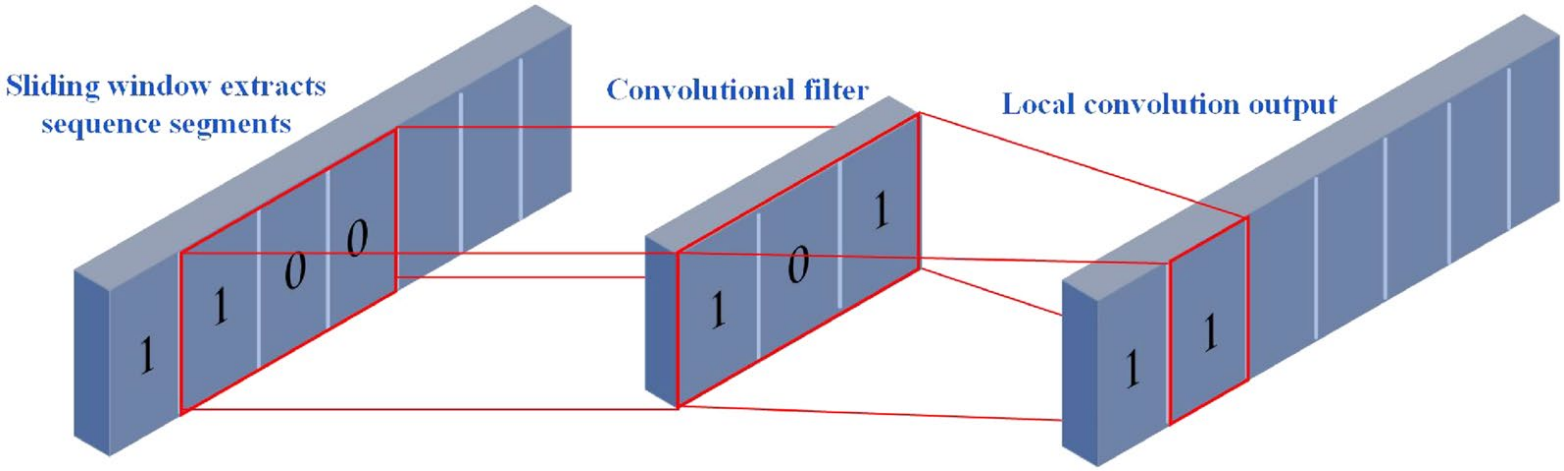
Schematic diagram of the convolution operation.

The convolution layer conducts a convolution operation between the input monitoring data and the convolution kernel to extract potential degradation features of engine performance. The fixed-size convolution kernel scans the entire data domain, resembling the visual perception of human eyes. Multiple convolution kernels with varying weights are employed to evaluate different aspects of each engine’s performance through convolutions, facilitating feature extraction. The schematic diagram illustrating the principle of convolution operation is presented in Fig. 2.

Each CNN layer contains several convolutional kernels with the same size and the same type of pooling function. The specific operation process of the convolutional layer and pooling layer is as follows: assume that the *l*th subsequence of the input is ${{x}_{l}}=\left[ {{x}_{l,1}},{{x}_{l,2}},\ldots ,{{x}_{l,N}} \right]$, where *N* denotes the length of the sequence. The convolution operation in the convolution layer can be defined as the multiplication operation between the convolution kernel ${{w}_{l}},{{w}_{l}}\in {{R}^{{{F}_{L}}}}$ and the connection vector ${{x}_{l,i:i+{{F}_{L}}-1}}$, which can be expressed as Eq. (1):

1 $$\begin{aligned} x_{l, i: i+F_L-1}=x_{l, i} \oplus x_{l, i+1} \oplus \cdots \oplus x_{l, i+F_L-1} \end{aligned}$$

where $x_{l, i: i+F_L-1}$ is a sampling window of the length $F_l$ of the *l*th subsequence starting from the *i*th point, and the symbol $\oplus$ concatenates each data sample into a longer embedding. The final convolution operation is defined as Eq. (2):

2 $$\begin{aligned} Y_l=\varphi \left( w_l^T x_{l, i-i+F_L-1}+b\right) \end{aligned}$$

where $w_l$ is the weight matrix for the *l*th kernel, $\varphi$ represents the selected nonlinear activation function, and *b* represents the bias term. The output $Y_l$ is the corresponding performance degradation feature extracted by the convolution operation on the *l*th subsequence $x_{l, i: i+F_L-1}$. By sliding the filtering window from the first point to the last point of the sample data, the *l*th degenerate feature obtained can be written as Eq. (3):

3 $$\begin{aligned} Y_l=\left[ Y_l^1, Y_l^2, \ldots , Y_l^{N-F_L+1}\right] \end{aligned}$$

The ReLU activation function, commonly employed in this study, is selected to perform nonlinear transformations on the output features of the convolutional layer. This choice aims to enhance network sparsity and mitigate overfitting risks when dealing with few-shot conditions.

The two common pooling operations in convolutional neural networks are MaxPooling and AveragePooling. Among them, the MaxPooling function is widely used due to its ability to enhance subtle features. Following the convolution and pooling operations, the size of each dimension of the output feature can be calculated using a formula, as shown in Eq. (4):

4 $$\begin{aligned} o=\frac{n+2 p-h}{s}+1 \end{aligned}$$

where *n* is the size of each dimension of the input feature, *p* is the size of the padding, *h* is the size of the convolution kernel, and *s* is the stride.

### Gated recurrent unit

**Figure 3:**
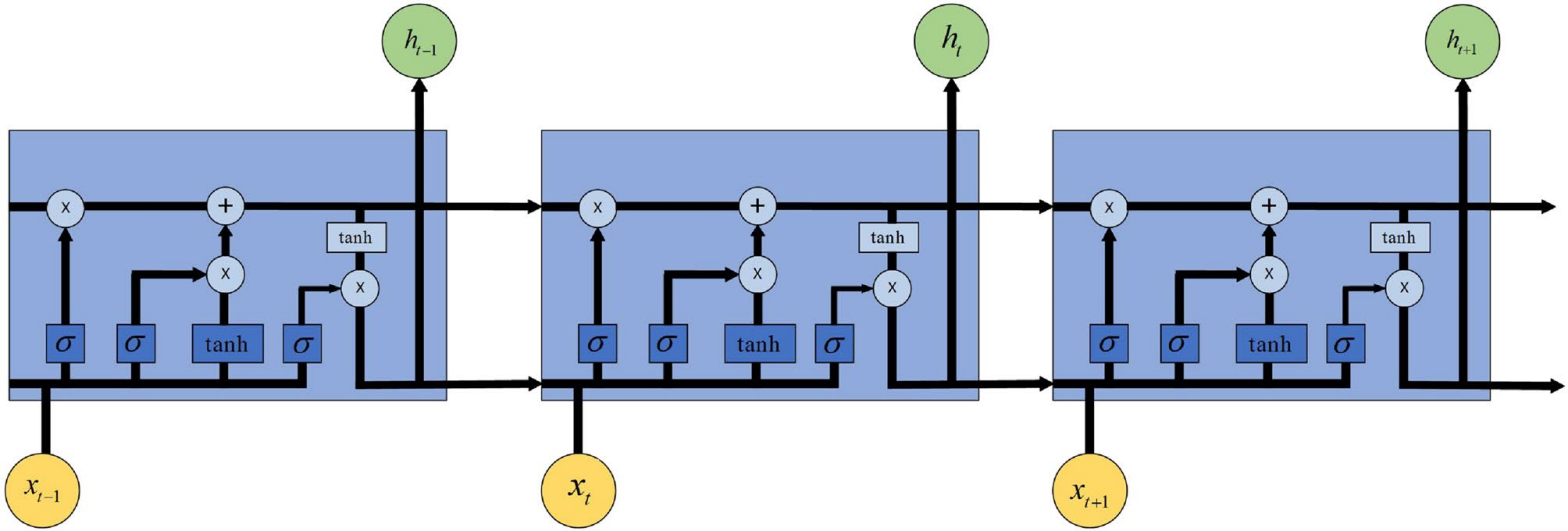
Basic structure of LSTM network.

In this paper, the input of GRU is formed by the feature sequence generated after convolution. GRU is a variant of LSTM that improves upon the RNN network. By introducing a gating mechanism to control information accumulation speed, new information can be selectively added while previous information can be forgotten. This addresses the issues of long-term dependence and gradient disappearance during long-sequence training in RNN. Therefore, this section briefly introduces the LSTM network as shown in Fig. 2. The repetition module of LSTM consists of four interactive layers with activation functions. $\sigma$ represents the sigmoid function which maps data to values between 0 and 1, while ’tanh’ function maps data to values between -1 and 1. The basic network structure is depicted in Fig. 3. The mathematical descriptions for sigmoid and tanh functions are provided in Eq. (5).

5 $$\begin{aligned} \begin{aligned}{}&\sigma (x)=\frac{1}{1+\textrm{e}^{-x}} \\&\tanh (x)=\frac{\textrm{e}^x-\textrm{e}^{-x}}{\textrm{e}^x+\textrm{e}^{-x}} \end{aligned} \end{aligned}$$

The network parameters are presented in Table 1.

**Table 1:** Meaning of basic parameters of LSTM network.

Notations	Description
$x_t$	Input information at the current
$f_t$	Forget gate control signal
$C_{t-1}$	Memory cells at the previous time
${{\tilde{C}}_{t}}$	Information candidate state
$i_t$	Input gate control signal
$C_t$	Memory cells at the current time
$h_{t-1}$	Hidden state at the previous time
$o_t$	Output gate control signal
$h_t$	Hidden state at the current time

**Figure 4:**
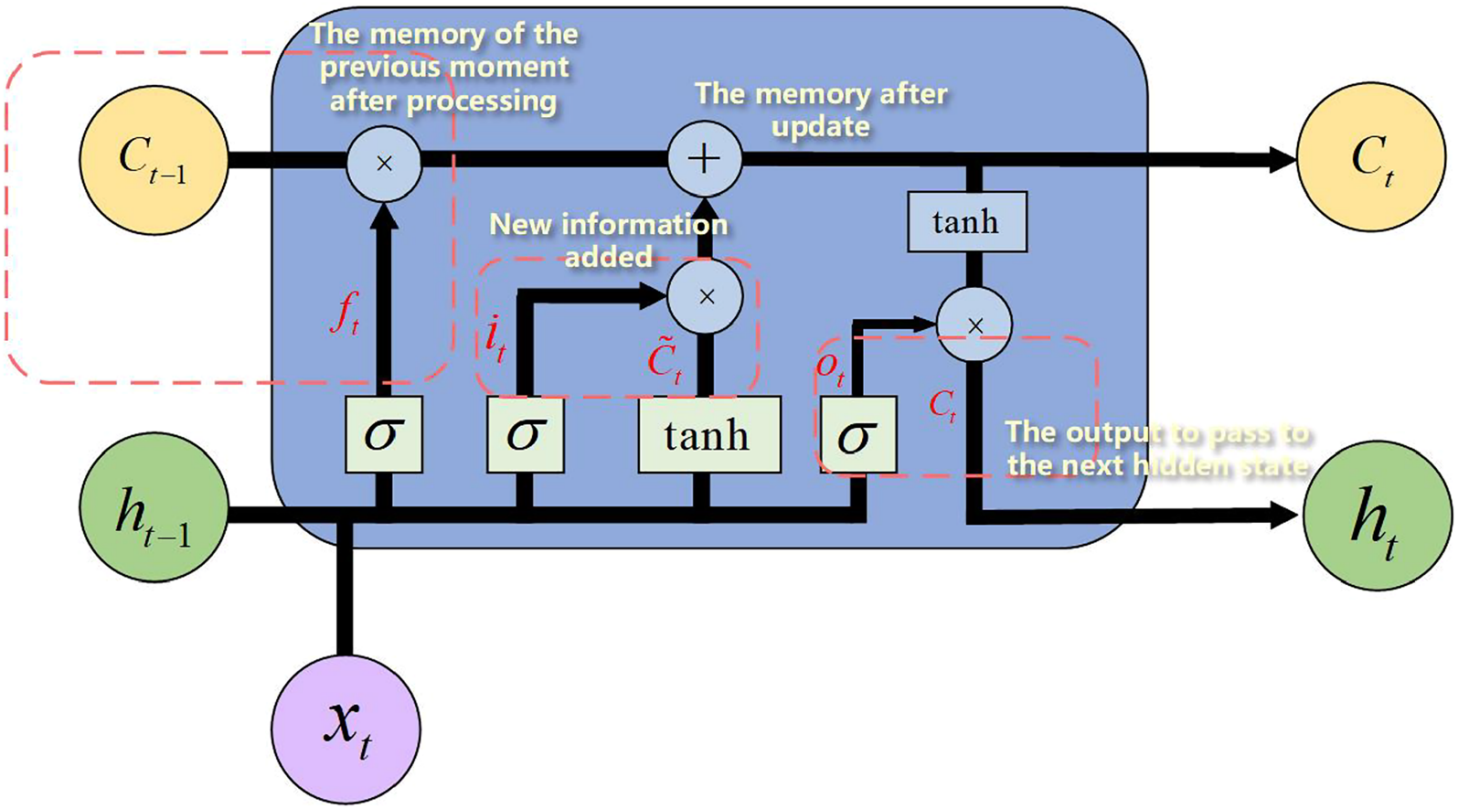
Memory update process of LSTM.

LSTM possesses three types of gate structures: forget gates, input gates, and output gates. The memory unit at the previous moment $C_{t-1}$ determines which information should be forgotten, retained, or unchanged through the control of three gates and linear operation, to generate the memory unit at the current moment $C_t$, and then output it to the next moment to update the memory. The specific update process is shown in Fig. 4.

The first step of LSTM involves controlling the forgetting factor of the previous time step’s information through the forget gate *f*, while the second step determines which new input information can be stored in the new memory cell through the input gate *i*. The memory cell is then updated by integrating signals from the forget gate and input gate. The third step regulates the amount of information from the current memory cell state that needs to be output to the hidden state $h_t$ through the output gate $o_t$, facilitating the transmission of new information to the next time step. The mathematical description of LSTM’s forward propagation process is represented as Eq. (6):

6 $$\begin{aligned} \begin{aligned} f_t&=\sigma \left( W_{f x} x_t+W_{f h} h_{t-1}+b_f\right) \\ i_t&=\sigma \left( W_{i x} x_t+W_{i h} h_{t-1}+b_i\right) \\ \tilde{C}_t&=\tanh \left( W_{\alpha x} x_t+W_{c h} h_{t-1}+b_c\right) \\ C_t&=f_t \odot C_{t-1}+i_t \odot \tilde{C}_t \\ o_t&=\sigma \left( W_{o x} x_t+W_{o h} h_{t-1}+b_0\right) \\ h_t&=o_t \odot \tanh \left( C_t\right) \end{aligned} \end{aligned}$$

where $\odot$ represents the Hadamard product, which is the element-wise multiplication of two matrices of the same type.

**Figure 5:**
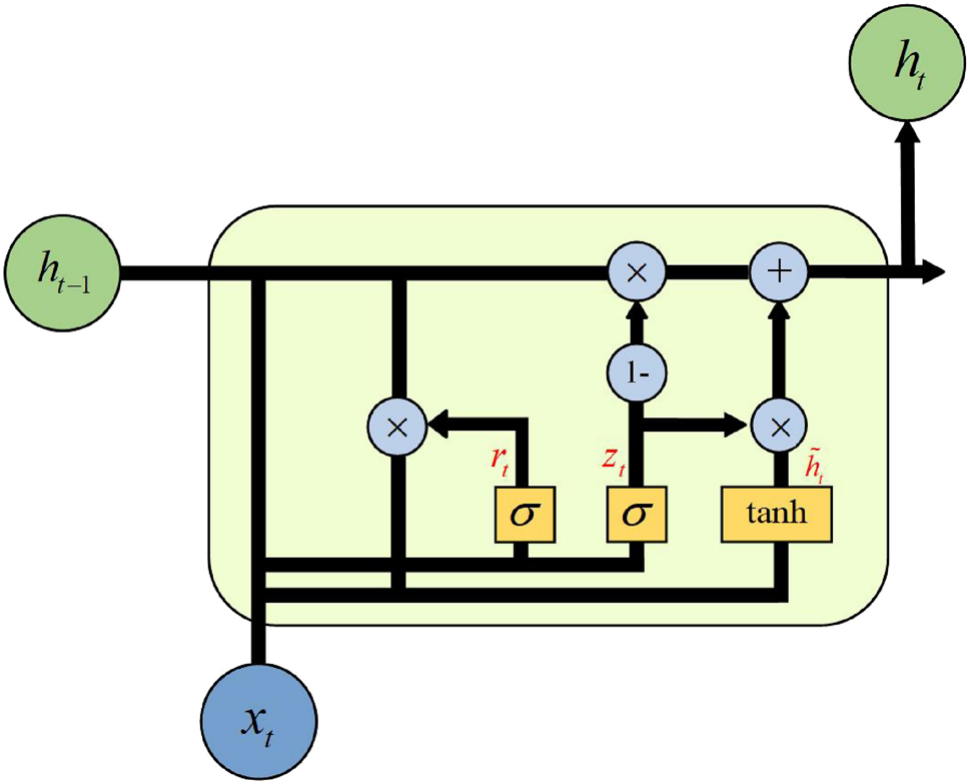
The gate unit structure of GRU.

In GRU, there are only two gates: the reset gate, which is equivalent to the forget gate and input gate, and the update gate. This simplified structure makes GRU more efficient and easier to train compared to standard LSTM models. The gate unit structure of GRU is illustrated in Fig. 5.

The network parameters are presented in Table 2.

**Table 2:** Meaning of basic parameters of GRU network.

Notations	Description
$x_t$	Input information at the current time
$z_t$	Update gate control signal
$r_t$	Reset gate control signal
${{\tilde{h}}_{t}}$	Candidate hidden states
$h_{t-1}$	Hidden state at the previous time
$h_t$	Hidden state at the current time

As shown in the table, $r_t$ determines how new input information is combined with the previous memory and controls the extent to which hidden state information from the previous moment $h_{t-1}$ is written to the current candidate state ${{\tilde{h}}_{t}}$. ${{z}_{t}}$ controls the extent to which information from the previous hidden state $h_{t-1}$ is brought into the current hidden state $h_t$. The mathematical description of GRU’s forward propagation process is represented as Eq. (7):

7 $$\begin{aligned} \begin{aligned} f_t&=\sigma \left( W_{f x} x_t+W_{f h} h_{t-1}+b_f\right) \\ i_t&=\sigma \left( W_{i x} x_t+W_{i h} h_{t-1}+b_i\right) \\ \tilde{C}_t&=\tanh \left( W_{\alpha x} x_t+W_{c h} h_{t-1}+b_c\right) \\ C_t&=f_t \odot C_{t-1}+i_t \odot \tilde{C}_t \\ o_t&=\sigma \left( W_{o x} x_t+W_{o h} h_{t-1}+b_0\right) \\ h_t&=o_t \odot \tanh \left( C_t\right) \end{aligned} \end{aligned}$$

Equation (7) shows that the output of GRU is the weighted sum of the output at the previous time $h_{t-1}$ and the candidate hidden state ${{\tilde{h}}_{t}}$. Through the adaptive recursive weighting mechanism, the GRU unit calculates recursive weighting coefficients based on the input at the current time step and the state at historical time steps. These coefficients are then applied to various parts of the network to adjust their influence or weights. This mechanism allows the network to handle information from different time steps more flexibly, highlighting important information for the current task or suppressing irrelevant information, thereby enhancing its capability to process sequential data more effectively.

### Prescreening method for monitoring data

In the initial feature pre-screening step, the primary objective is to identify the most sensitive features that contain sufficient degradation information. To ensure that good degradation features possess significant time correlation and exhibit monotonic changes with the development of degradation, this paper incorporates feature selection criteria proposed by Wu et al.^22^, and preliminarily screens multi-source sensor monitoring data using two indicators: a correlation matrix and a monotonicity matrix.

Most traditional feature selection methods are more suitable for classification problems. However, for remaining life prediction in time series data, deep learning models like CNN-GRU have stricter requirements for high-quality data representation, where traditional feature selection methods may not fully consider complex relationships between features and models. Correlation and monotonicity indicators directly impact input data quality by aiding accurate learning of relationships between features by models while effectively reducing unnecessary noise and redundant information in data. Consequently, this reduces overfitting risks during model training processes-particularly crucial for deep learning models with limited sample sizes.

The correlation matrix reflects the relationship between feature dimensions and running time, while the monotonicity matrix represents trends in features along the time dimension. This selection method guarantees that selected feature dimensions contain richer degradation information, thereby reducing computational complexity and improving prediction model performance. The *i*th column in the original feature matrix represents the time series feature sequence monitored by the *i*th sensor, while the correlation index with time is computed as follows:

8 $$\begin{aligned} {\text {Corr}}_i=\frac{\left| \sum _{t=1}^T\left( x_t^{(i)} -\bar{x}^{(i)}\right) (t-\bar{t})\right| }{\sqrt{\left| \sum _{t=1}^T\left( x_t^{(i)}-\bar{x}^{(i)}\right) ^2 \sum _{t=1}^T(t-\bar{t})^2\right| }} \end{aligned}$$

where $x_t^{(i)}$ represents the *i*th observation at time step *t*, while $\bar{x}^{(i)}$ and $\bar{t}$ respectively represent the average values of ${{x}^{(i)}}$ and *t* over the entire period time.

The formula for computing monotonicity indicators is as follows:

9 $$\begin{aligned} \textrm{Mono}_i=\left| \frac{d x^{(i)}>0}{T-1}-\frac{d x^{(i)}<0}{T-1}\right| \end{aligned}$$

where *T* represents the length of the sample in the life cycle, $d{{x}^{(i)}}$ represents the difference of ${{x}^{(i)}}$.

The aforementioned two metrics exhibit values within the range of [0,1] and demonstrate a positive correlation with the sensitivity of the feature. Subsequently, a weight coefficient $\alpha$ is introduced to compute the linear combination of these two indicators as the ultimate criterion for feature selection, as depicted in Eq. (10):

10 $$\begin{aligned} {\text {Cri}}_i=\alpha \cdot {\text {Corr}}_i+(1-\alpha ) \cdot \textrm{Mono}_i \end{aligned}$$

where $\alpha$ ranges from [0,1] and represents the coefficient that effectively balances the contributions of the two metrics in the feature selection.

## Proposed method

**Figure 6:**
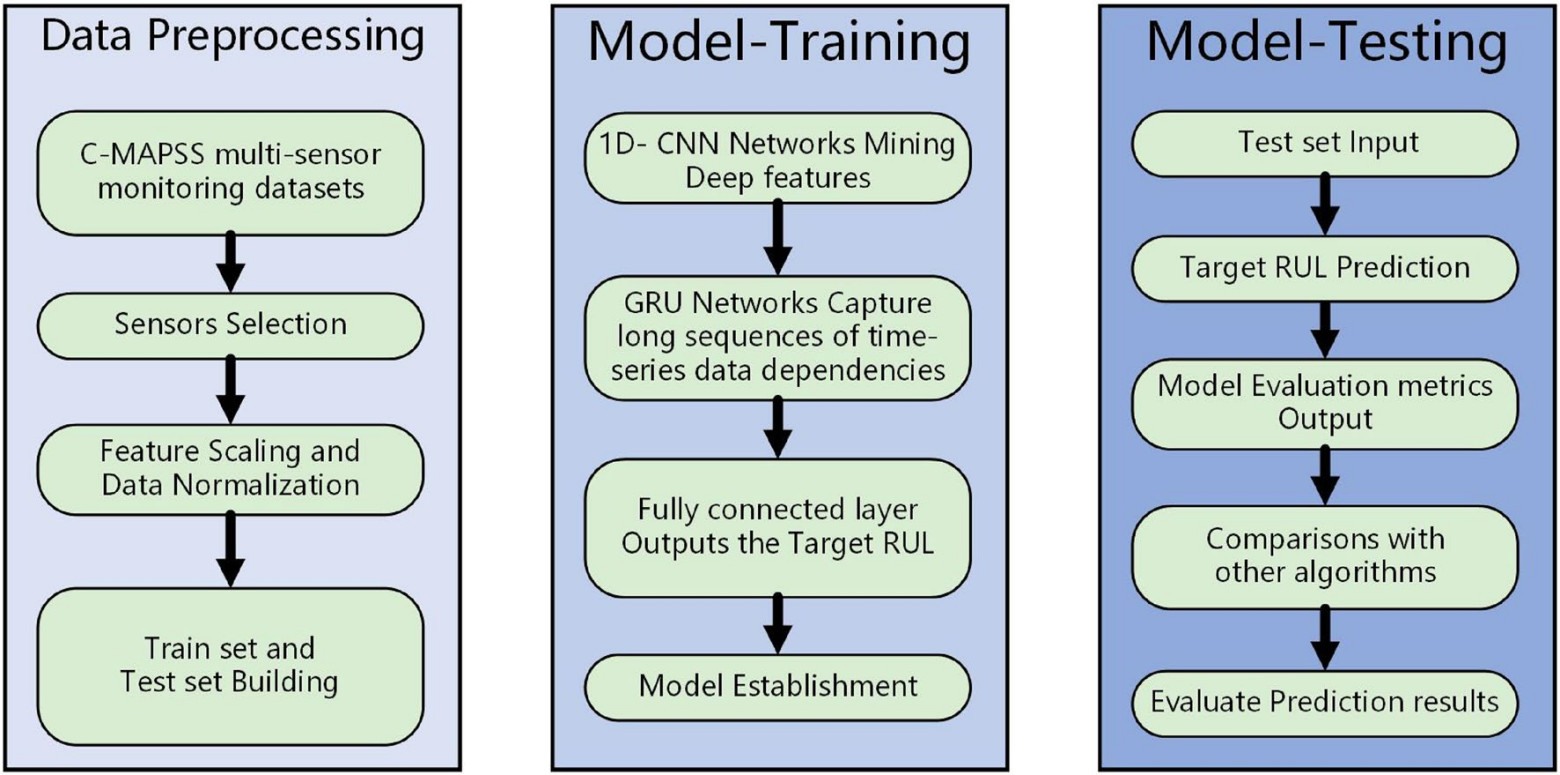
Overall architecture of the proposed model.

In this study, we propose a prediction model based on a hybrid network of 1D CNN-GRU for RUL prediction in complex systems with few-shot conditions. In the CNN architecture employed in this study, convolutional and pooling layers play a pivotal role. The convolutional layer applies a series of filters through a sliding window to capture local features in the input degradation monitoring data, effectively identifying and extracting time-dependent features such as periodic changes, trends, and mutation points. Moreover, it preserves the spatial structure information of the data while extracting features at various spatial scales by combining multiple convolution kernels, thereby capturing a more comprehensive and abstract representation of the data. The pooling layer aggregates these features using Max pooling operation to reduce the spatial dimensionality of the data. This dimension reduction aids in decreasing computational complexity while enhancing model robustness and generalization ability towards diverse datasets. Simultaneously, it ensures model stability and prediction consistency by making the model invariant to minor variations in input data. In RUL prediction tasks specifically, pooling layers facilitate capturing crucial characteristic patterns within time series. The convolutional and pooling layers in summary provide a more efficient representation of input data for the GRU layer by extracting and processing features within the CNN architecture. Their synergy is primarily manifested in feature abstraction, identification, temporal dependency modeling, and integration of multi-scale features. By leveraging convolutional layers and pooling layers, the GRU layer effectively extracts integrated feature representations to facilitate higher-level temporal dependency modeling. The proposed model consists of three stages, namely data preprocessing, training stage, and prediction stage. The overall architecture is illustrated in Fig. 6.

The specific methodology is outlined as follows:

- Preprocessing of the dataset: First, select the monitoring parameters of the complex system. Next, use MinMaxScaler to scale the test data features, scaling them to the specified feature range. Generate uniform noise and add it to the feature-scaled data. Randomly shuffle the training dataset to increase the diversity of model training.
- After preprocessing the data, the feature matrices obtained from applying a sliding time window are organized into standardized training, validation, and test datasets for the deep mixture model. The model is trained using the data from the training set while monitoring its fitting performance on the validation set.
- The sensor data processed in step one serves as the input for the two-layer CNN. The CNN layer conducts convolution operations between the input sample data and a one-dimensional convolution kernel, extracting intrinsic performance degradation features contained within the data through an activation function that incorporates a bias term. This enables comprehensive and profound exploration of potential features.
- The deep features extracted from last step are input into the GRU network, and the unique memory unit structure in the GRU network is used to capture the time series correlation of data features, so as to successfully establish a prediction model.
- In the training phase, the training dataset is randomly divided into batches, employing Rectified Linear Unit (ReLU) as the activation function and Mean Square Error (MSE) as the loss function. To mitigate overfitting during model calculation, Dropout operations are incorporated in both the CNN layer and the fully connected layer of the deep mixture model.
- Hyperparameters are optimized using the cross-validation method. Once the model completes a specified number of training iterations, it evaluates whether the output results meet the desired level of accuracy. If not, it adjusts the parameter values and continues training until the desired accuracy is achieved. If so, it outputs the final result.
- In the prediction stage, the trained deep mixture model is utilized to estimate the real-time RUL of each object based on the test dataset. Subsequently, the model’s predictive performance index was employed to evaluate the accuracy and generalization capability by comparing its predicted values against the actual RUL values from the test set. Furthermore, a comparative analysis with other relevant algorithms was conducted

### CNN-GRU network structure

**Figure 7:**
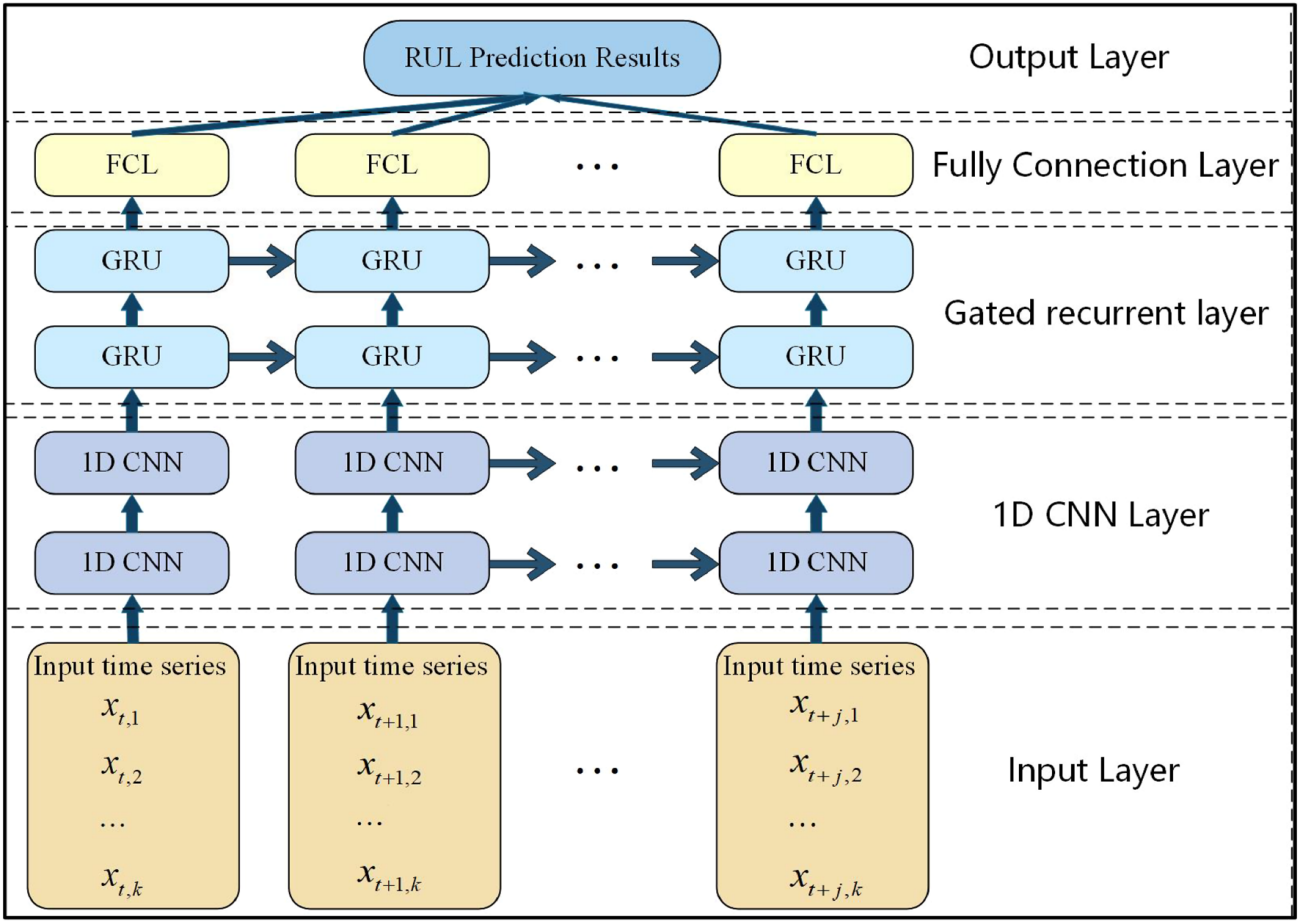
The proposed CNN-GRU network.

The CNN-GRU network architecture utilized in this study is illustrated in Fig. 7., comprising an input layer, a CNN layer, a GRU layer, a fully connected layer, and an output layer. In order to address the challenges of overfitting and insufficient data mining in small sample time series data, we proposed specific enhancements to the GRU component, as described below: Firstly, the parameters of the gating mechanism are adapted to better capture patterns and changes in the degradation time series of the selected FD001 dataset. Additionally, by increasing the depth of the GRU network and stacking multi-layer GRU units, high-level feature representations of time series data are abstracted and extracted step by step, thereby improving the generalization ability and prediction accuracy of the model for small samples. Besides, regularization and batch normalization techniques are introduced to enhance the performance of the GRU module. The application of regularization techniques such as Dropout and Batch Normalization helps reduce overfitting risks during training while accelerating convergence.

In the RUL prediction task, sliding window sampling can capture more temporal correlation information from time series data compared to single-time step sampling. Therefore, firstly, at each time step, the selected sensor data is collected to form a high-dimensional feature vector that serves as the input of the network. Then, the multi-layer CNN uses the weights on the convolutional layers to realize parameter adjustment and automatically extract high-level abstract features. It is worth noting that the neurons in the middle employ the Dropout method to prevent the network from overfitting, and the Max pooling layer is used for feature dimensionality reduction. Next, the abstract feature sequence extracted by CNN is input into the GRU layer, and the GRU is used to combine the features at different time points of the sequence to fully learn the temporal relationship of the features. The final deep feature representation is generated in the hidden layer and input into the activation function for optimization. Finally, the prediction results were directly output by the linear regressor of the fully connected layer to complete the RUL prediction of complex systems under the few-shot conditions

### Selection of loss function and optimization algorithm

When training the CNN-GRU neural network, the optimization loss function ${{L}_{loss}}$ employed is the MSE, which is commonly utilized in traditional regression problems as depicted in Eq. (11).

11 $$\begin{aligned} L_{\text{ loss } }=\frac{1}{N} \sum _{i=1}^N\left( y_i-\hat{y}_i\right) ^2 \end{aligned}$$

where $y_i$ and ${{\hat{y}}_{i}}$ respectively represent the true and predicted values of RUL, and *N* represents the total number of samples in the training set. Adaptive Moment Estimation (Adam) optimizer has been selected as the model parameter optimization algorithm.

During the training process, by visualizing the trend of the loss function on the training set and the test set for each round of model training, we found that the model was suffering from overfitting. To solve this problem, a regularization term $L_2$ was introduced into the model, as shown in Eq. (12):

12 $$\begin{aligned} J(w, b)=\frac{1}{m} \sum _{i=1}^m L\left( \hat{y}^{(i)}, y^{(i)}\right) +\frac{\lambda }{2 m}\Vert w\Vert _2^2 \end{aligned}$$

In the context of few-shot for deep learning prediction modeling, the selection and adjustment of the learning rate play a crucial role. Recognizing that an appropriate learning rate facilitates faster convergence to the optimal solution while avoiding local optima or oscillation, this study employs a dynamic learning rate strategy. By incorporating a learning rate scheduler, we initialize the learning rate with a higher value in preceding batches and subsequently attenuate it in subsequent batches.

## Experiments and results

### Datasets and evaluation metrics

**Figure 8:**
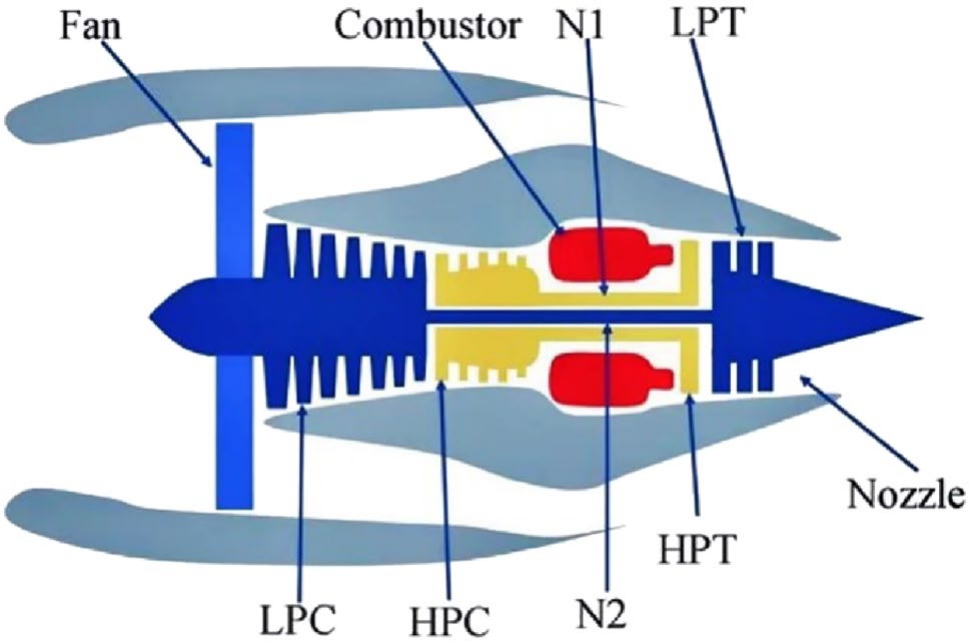
Schematic diagram of turbofan engine structure.

The dataset used in this paper is the publicly available turbofan degradation monitoring dataset provided by NASA. Due to the complex structure and diverse airflow patterns of aircraft engines, obtaining their operational data is usually difficult and confidential. Therefore, NASA generated this simulation dataset using the Commercial Modular Aero-Propulsion System Simulation software, called C-MPASS for short, aiming to evaluate the performance of different models by combining the characteristics of engine operation. The dataset includes the main components such as the fan, low-pressure compressor (LPC), high-pressure compressor (HPC), combustor, high-pressure turbine (HPT), low-pressure turbine (LPT), and nozzle. The schematic diagram of its structure is shown in Fig. 8.

Over time, every engine will inevitably encounter a failure event, resulting in the termination of sensor data collection. Consequently, the determination of the actual RUL relies on the duration of available data. This dataset was previously employed as challenge data for the inaugural Forecasting and Health Management (PHM) Data Competition in 2008, which has certain experimental values.

The C-MAPSS dataset comprises four sub-datasets, each with varying numbers of operating and fault conditions. Furthermore, each sub-dataset is further divided into training and test subsets. The training subset captures the sampled values of multiple state parameters for the aero-engine throughout its complete cycle from normal to faulty operation. Conversely, the test subset contains state parameter values leading up to a specific time point before failure along with their corresponding remaining life expectancy. Each row in the dataset represents a snapshot of data collected during a single runtime period. This comprehensive dataset consists of 26 columns: unit number (column 1), current operating cycle number (column 2), three monitoring parameters for operational settings (Flight altitude, Mach number, Throttle lever angle) represented by columns 3–5 respectively, and sensor measurement values (columns 6–26) encompassing measurements from 21 sensors installed on each engine that capture various aspects related to engine condition during operation. Its 24 sensor monitoring parameters and physical meanings are shown in the following Table 3.

**Table 3:** Sensor monitoring parameters.

Id	Notations	Physical connotation
1	*H*	Flight altitude
2	*Ma*	Mach number
3	*TRA*	Throttle lever angle
4	*T*2	Fan inlet temperature
5	*T*24	Outlet temperature of low-pressure compressor
6	*T*30	Outlet temperature of high-pressure compressor
7	*T*50	Outlet temperature of low-pressure turbine
8	*P*2	Fan inlet pressure
9	*P*15	Total external bypass pressure
10	*P*30	Total outlet pressure of high-pressure compressor
11	*NF*	Uncorrected fan speed
12	*NC*	Uncorrected core machine speed
13	*EPR*	Engine pressure ratio
14	*PS*30	Static pressure at the outlet of the high-pressure compressor
15	*PHI*	Fuel flow and P30 ratio
16	*NRF*	Corrected fan speed
17	*NRC*	Corrected core machine speed
18	*BPR*	Bypass ratio
19	*FARB*	Combustion chamber gas ratio
20	$HT\_BLEEd$	The enthalpy of bleed air
21	$NF\_DMD$	Fan speed command value
22	$PCNFR\_DMD$	Fan correction speed command value
23	*W*31	The high-pressure turbine cooling airflow
24	*W*32	The low-pressure turbine cools the airflow

To better align with the few-shot conditions, this study utilizes a subset of the FD001 simulation data. Specifically, it includes only 100 complete engine simulations from initial operation to failure, where each time sequence starts with normal engine functioning and ends with an unknown failure occurrence. In the training set, fault magnitude progressively increases until system failure is observed. The test set provides data up to a certain point before system failure. The objective is to estimate the remaining number of operating cycles before test data failure.

To validate the predictive performance of the proposed algorithm, commonly employed evaluation metrics in the field of remaining useful life prediction, namely Root Mean Square Error (RMSE), Coefficient of Determination ${{R}^{2}}$, and S_Score are utilized as assessment indicators. The calculation formulas for these metrics are provided as follows:

13 $$\begin{aligned} R M S E&=\sqrt{\frac{1}{N} \sum _{i=1}^N\left( y_i-\hat{y}_i\right) ^2} \end{aligned}$$

14 $$\begin{aligned} R^2&=1-\frac{{\text {SSR}}}{{\text {SST}}} =1-\frac{\sum _{i=1}^N\left( y_i-\hat{y}_i\right) ^2}{\sum _{i=1}^N\left( y_i-\bar{y}\right) ^2} \end{aligned}$$

15 $$\begin{aligned} S\_Score&=\left\{ \begin{array}{l} \sum _{i=1}^N\left( \textrm{e}^{-\frac{\hat{y}_i-y_i}{13}}-1\right) , y_i>\hat{y}_i \\ \sum _{i=1}^N\left( \textrm{e}^{\frac{\hat{y}_i-y_i}{10}}-1\right) , y_i \leqslant \hat{y}_i \end{array}\right. \end{aligned}$$

where $y_i$ and ${{\hat{y}}_{i}}$ represent the real and predicted remaining life value, respectively, and *N* represents the total number of samples in the test set. and SSR (Sum of Squares Residual) is the sum of squares of residuals, representing the sum of differences between the predicted value of the model and the actual observed value. Total Sum of Squares(SST) represents the total variation in the predicted values, i.e. the sum of the differences between the actual observed values and the predicted mean. The S_Score is a measure of the accuracy of a prediction model in predicting remaining useful life, and the scoring function has been proposed by many researchers^23,24^, and adopted by the International Conference on Prognostics and Health Management Data Challenge. It takes into account the deviation between the predicted value and the actual value and converts and accumulates the deviation using an exponential function, it is worth noting that S_Score penalizes later predictions more than early predictions, because later predictions often lead to more serious consequences in many fields such as the aerospace industry. For this experiment, the lower the values of RMSE and S_Score are, the closer ${{R}^{2}}$ is to 1, indicating that the model’s prediction accuracy is higher.

### Experimental settings

The programming language used in this experiment is Python 3.8, while the deep learning framework for the prediction model is based on TensorFlow, PyTorch, and Scikit-learn, among others. The Seaborn library is employed as the data visualization tool, and Matplotlib plotting module serves as the plotting tool.

To identify overfitting and underfitting, multiple models are evaluated for their performance during training, with timely adjustments made to hyperparameters. The training set is divided into a training set and a validation set.

Regarding the preprocessed data, the training data consists of 14,184 time windows. Each window corresponds to 30 time steps with 12 feature values per step (shape: (14,184, 30, 13)). The validation data has a shape of (3547, 30, 13), while the test data has a shape of (497, 30 ,13). As for processed real RULs, they are represented by one-dimensional arrays that match each corresponding time window’s length.

Subsequently, considering the inherent issue of diverse sensor types within the turbofan system, each with distinct working conditions and numerical ranges, this study employs the classical Min-Max normalization technique to preprocess the input sensor data. This preprocessing step aims to rescale the sensor monitoring data within a range of [0,1], thereby facilitating the effective processing of data from disparate sensors by the model and enhancing both neural network fitting speed and overall prediction accuracy. Simultaneously, in order to enhance model generalization ability and mitigate overfitting during training, this paper introduces noise perturbation to augment diversity in input sensor data as well as bolster model robustness. Specifically, the calculation formula is expressed as Eq. (16):

16 $$\begin{aligned} \begin{aligned} \tilde{z}^{(i)}=\frac{z^{(i)}-\min \left( z^{(i)}\right) }{\max \left( z^{(i)}\right) -\min \left( z^{(i)}\right) } \\ z^{(i)}=x^{(i)}+p^{(i)} \end{aligned} \end{aligned}$$

where $p^{(i)}$ represents the uniformly distributed noise introduced to the *i*th sensor.

We conducted a parameter sensitivity analysis through multiple iterations, exploring various values for the learning rate (lr), Dropout ratio of CNN layer and fully connected layer (drop_CNN and drop_dense), as well as convolution kernel size (kernel_size), among other parameters. For each set of parameter configurations, we trained the model and evaluated its prediction performance (RMSE) on the test set. These parameters were systematically fine-tuned, with careful documentation of their specific impact on model performance. After stepwise optimization and cross-validation, we identified the best combination of parameters and recorded their corresponding RMSE values. This approach not only facilitated the discovery of an optimal model configuration but also ensured consistent and reliable predictive performance across diverse settings. Finally, we saved several of the best models. Take the best model as an example to analyze:

In the experiment, the data is initially fed into a one-dimensional Convolutional Neural Network (Conv1D) layer with 128 filters. Each filter has a convolution kernel size of 3 and uses a sliding sampling window stride of 30 with ’same’ padding to ensure complete processing of the convolution kernel data. The Rectified Linear Unit (ReLU) activation function is chosen for the convolution layer. Following feature extraction, the data undergoes pooling to enhance its features using the MaxPooling1D strategy with a pool size of 2. The extracted features from both the convolution and pooling layers are then inputted into GRU layer, where both CNN and GRU layers consist of 128 neurons each. To prevent overfitting, a Dropout layer is added between the CNN and Dense layers with dropout rates set at 0.1 and 0.2 respectively; meaning that there is a retention probability of 90% for neuron nodes in the CNN layer and an 80% retention probability for neuron nodes in the fully connected layer. Finally, the data is flattened into one dimension using Flatten layer before being inputted into Dense layers—Dense1 employs ReLU activation function with a size set at 5 while Dense2 utilizes a linear activation function with a size set at 1. Table 4 presents detailed information on network parameters for each respective layer obtained through experimental cross-validation optimization.

**Table 4:** CNN-GRU network structure settings.

*Layer*	Output Shape	Parameters
*Conv1D*	(30, 128)	5120
*Dropout*	(30, 128)	
*Pooling*	(15, 128)	
*Conv1D*	(15, 128)	49,280
*Dropout*	(15, 128)	
*Pooling*	(8, 128)	
*Flatten*	(8, 128)	
*GRU*	(8, 128)	99,072
*GRU*	(None, 128)	99,072
*Dense*	(None, 200)	25,800
*Dropout*	(None, 200)	
*Dense*	(None, 1)	201

During the construction and training of the CNN-GRU model, certain hyperparameters must be defined, such as the size of the temporal moving window, number of hidden units, dimensions of the convolutional kernel and filter, and batch size. To determine optimal values for these hyperparameters, we performed multiple trainings and cross-validation on the training set to identify the most effective combination (refer to Table 5). For optimization using Adaptive Moment Estimation (Adam), we assigned exponential decay rates ${{\rho }_{1}}$ and ${{\rho }_{2}}$ of 0.9 and 0.999 to estimate first-order and second-order matrices respectively.

**Table 5:** CNN-GRU network structure settings.

Hyperparameter	Value
Batch size	128
Epoch	30
Kerner size	3
Initial learning rate	0.001
Droprate CNN	0.1
Droprate dense	0.2
Time moving window size	30
Pooling size	2

### Monitoring parameters selection

Utilize Python’s Matplotlib library for visualizing the original monitoring data to gain a more comprehensive and intuitive understanding of its characteristics. For the FD001 sub-dataset, the training set Train_FD001.txt comprises 100 engine parameter information of engines that have completed their full lifecycle; whereas, the test set Test_FD001.txt includes 100 engine parameter information of engines that have not yet reached their full lifecycle. Additionally, RUL_FD001.txt records the true RUL of these 100 engines. Each engine’s parameter information encompasses three types of operational monitoring parameters (Flight altitude, Mach number, and Throttle lever Angle), along with 21 performance monitoring parameters. It is important to note that FD001 represents a single-condition dataset where the three operational monitoring parameters—flight altitude, Mach number, and throttle lever angle—remain relatively constant during the cruise period and are therefore excluded from consideration.

**Figure 9:**
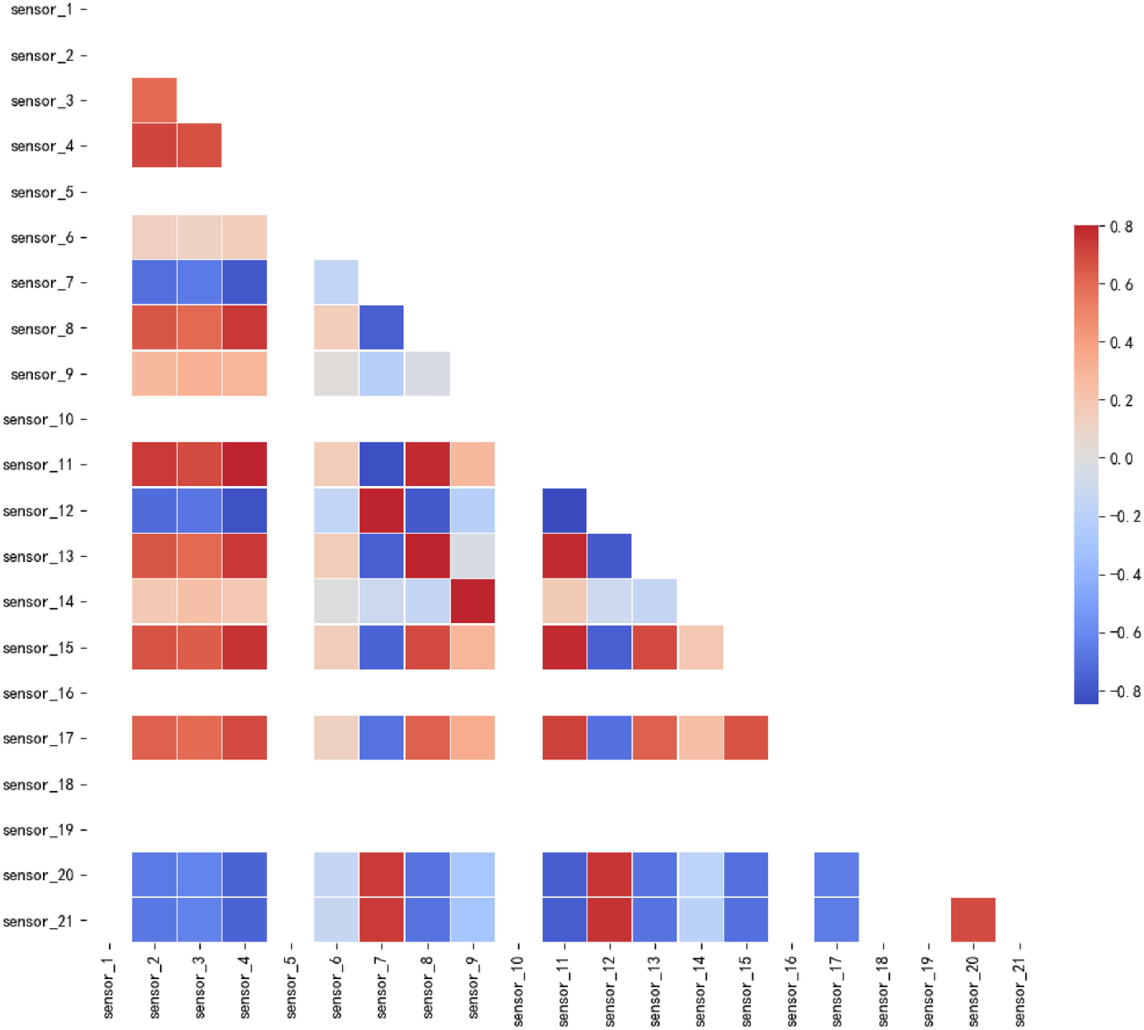
The heat map of sensor signal correlation.

After analyzing, due to the interrelated parts of the aircraft engine and the similar signal degradation process of some parts, as well as constant and discrete variables, the sensor signals are both correlated and uncorrelated. Therefore, there is both correlation and independence between variables. First, a heat map of the correlation between all engine sensor data is plotted in Fig. 9. This heat map shows the positive and negative correlations between variables, with red indicating a stronger positive correlation and blue indicating a stronger negative correlation. By observing the chart, the following conclusion can be drawn: some sensor information has an extremely high correlation, which may lead to redundant computational complexity; therefore, some sensor information can be deleted for further analysis. Additionally, some white columns may represent constant values, such as 1, 5, 10, 16, 18, and 19, which can be verified through other visualization operations.

A histogram containing the data distribution of 21 sensors was plotted as follows:

**Figure 10:**
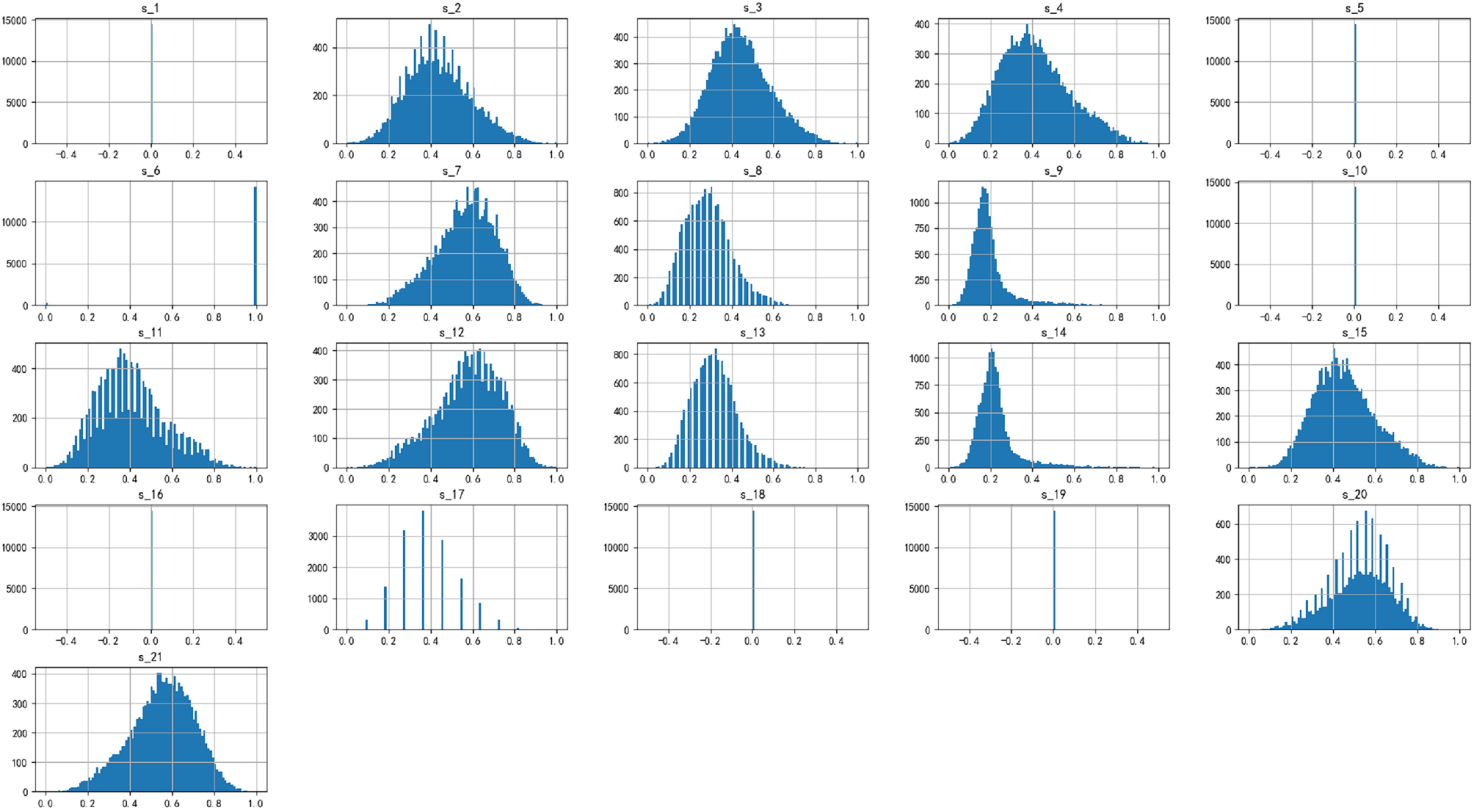
Histogram of sensor signal data distribution.

**Figure 11:**
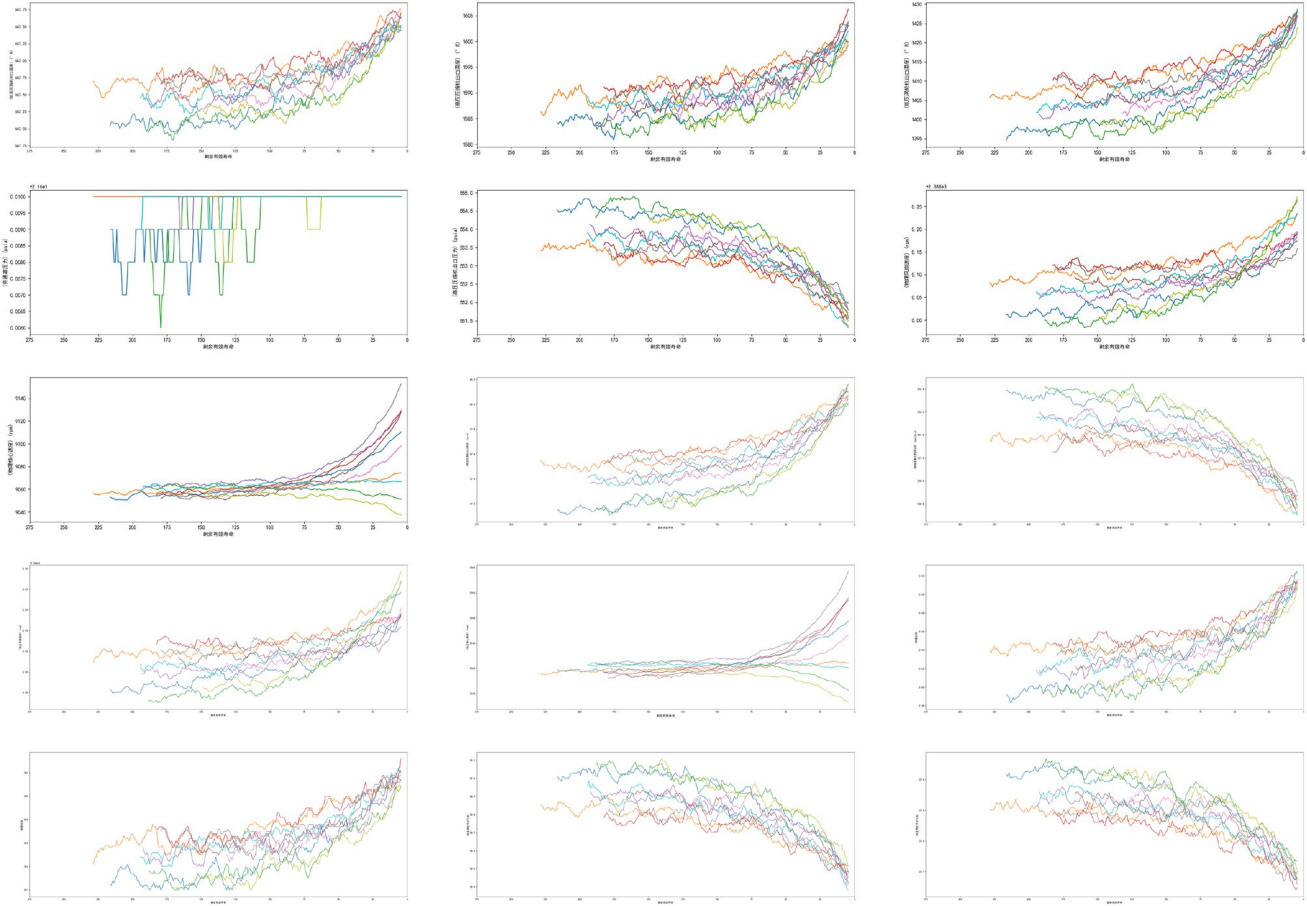
Trend chart of sensor data.

From Fig. 10, it can be observed that the data of sensors 1, 5, 6, 10, 16, 18, and 19 remain constant, while the data of sensor 17 exhibits a trend of discretization rather than continuity. Therefore, in the model training, we consider deleting these sensor data and retaining only the variables with continuous changes. Furthermore, almost all variables conform to the characteristics of univariate skewed Gaussian distribution, which is suitable for time series analysis and prediction modeling. Next, we visualized the trend of sensors 2, 3, 4, 6, 7, 8, 9, 11, 12, 13, 14, 15, 17, 20, and 21 changing with the remaining service life using Fig. 11, and used the Spearman rank correlation coefficient to calculate the correlation between the sensor data and the time cycle to determine its monotonicity. The results show that sensor 9, which exhibits a more significant monotonic trend than sensor 14, has a very high correlation, so we decided to retain sensor 9.

Finally, 12 sensor measurements from among the 21 sensors were selected as the original input features, with the corresponding serial numbers being ‘2’, ‘3’, ‘4’, ‘8’, ‘9’, ‘11’, ‘12’, ‘13’, ‘15’, ‘17’, ‘20’, and ‘21’. This is consistent with the results of the visual analysis mentioned earlier.

### Experimental results

**Figure 12:**
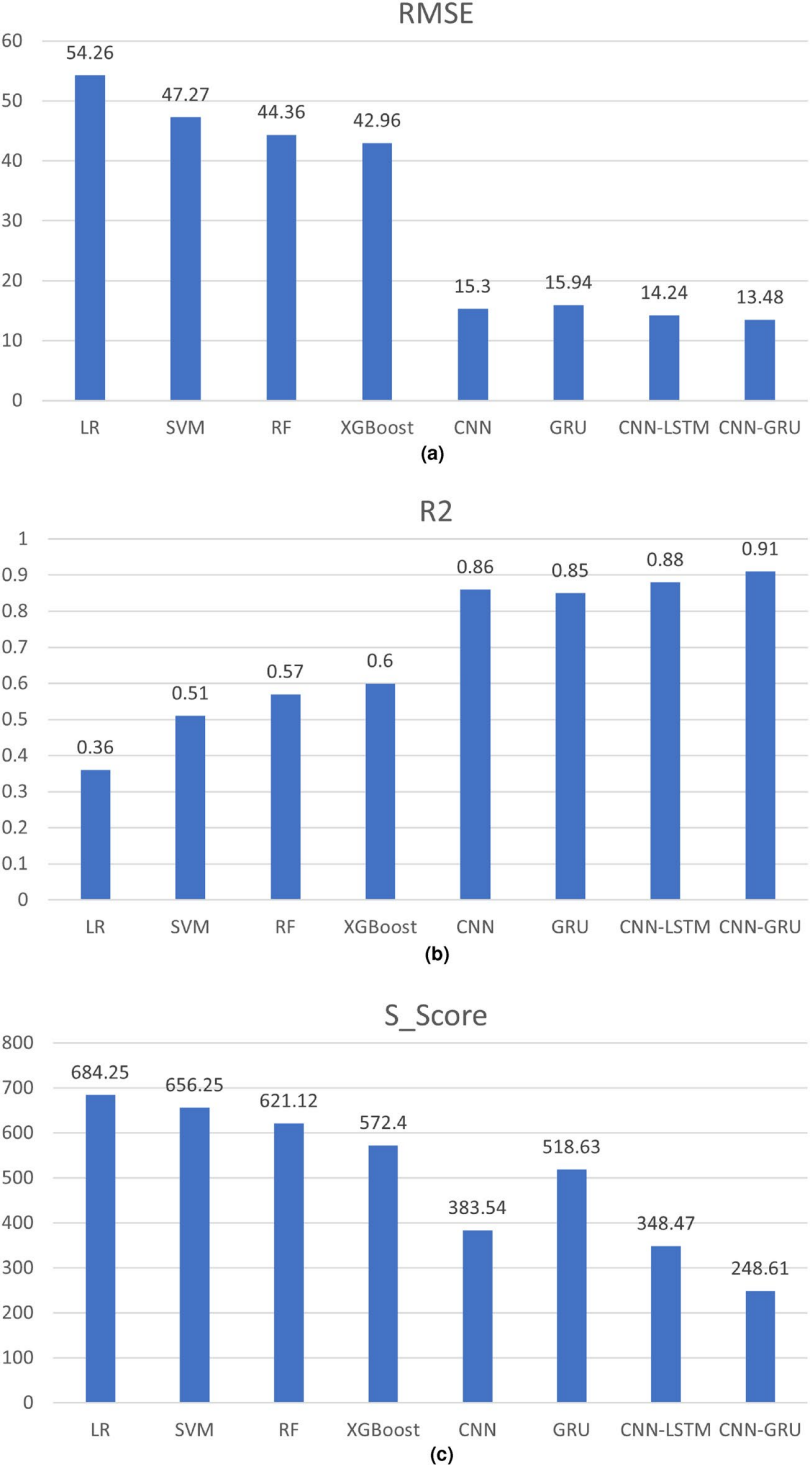
Comparison of prediction performance among different methods.

To verify the prediction performance of the proposed model under the few-shot conditions, we have compared its predictions with those of the Linear Regression (LR), Support Vector Machine (SVR), Random Forest (RF), Extreme Gradient Boosting (XGBoost), CNN, GRU, and CNN-LSTM models after parameter optimization, and presented the comparison of evaluation indicators in Fig. 12.

It can be observed that in comparison to traditional straightforward models such as LR, SVM, RF, and XGBoost, the proposed CNN-GRU model demonstrates a considerable and material enhancement in predictive prowess. Its RMSE has been diminished by 75%, 71%, 70%, and 69%, while its ${{R}^{2}}$ has been augmented by 153%, 78%, 60%, and 52%. This is attributed to the C-MAPSS dataset comprising a multitude of features and nonlinear correlations, rendering traditional models insufficient in capturing intricate feature relationships. In contrast to employing deep learning models CNN and GRU independently, the CNN-GRU model reduces RMSE by 12 and 13.6%, enhances ${{R}^{2}}$ by 6% and 7%, and lessens S_Score by 35% and 52%. When compared with the CNN-LSTM model, the CNN-GRU model brings down RMSE by 5%, improves ${{R}^{2}}$ by 3.4%, and curtails S_Score by 29%.

**Figure 13:**
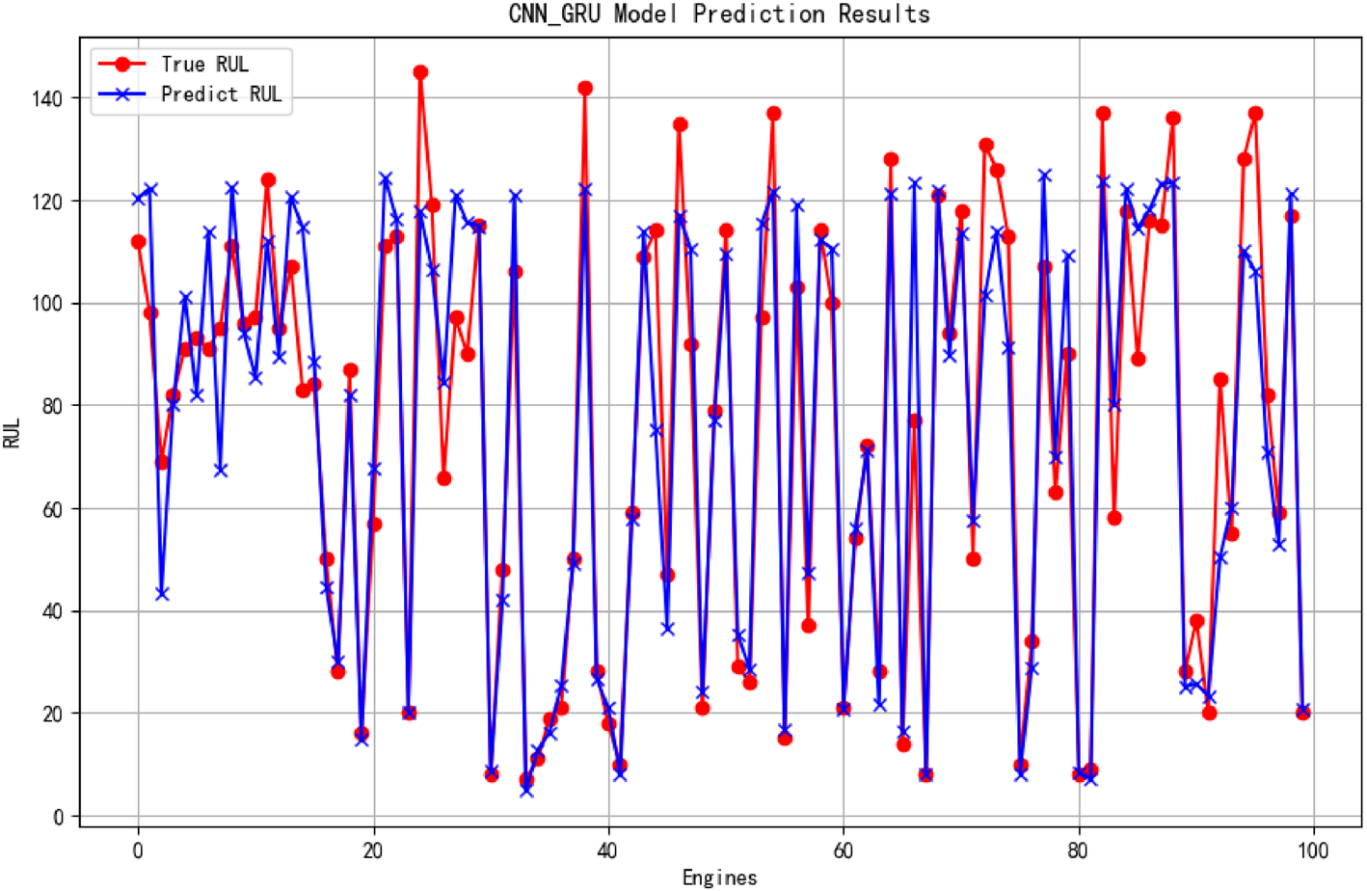
The result of RUL prediction.

**Figure 14:**
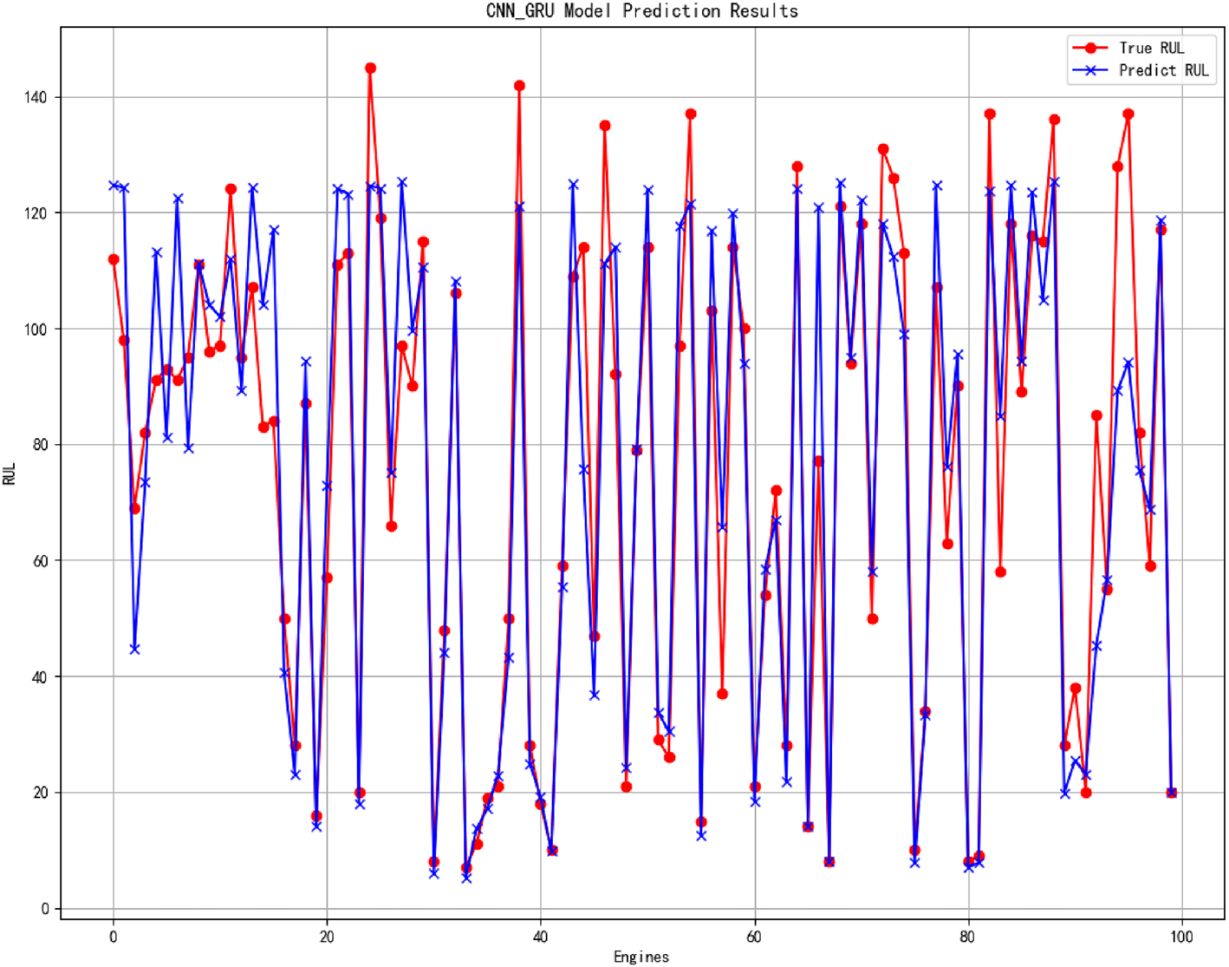
The result of RUL prediction.

**Figure 15:**
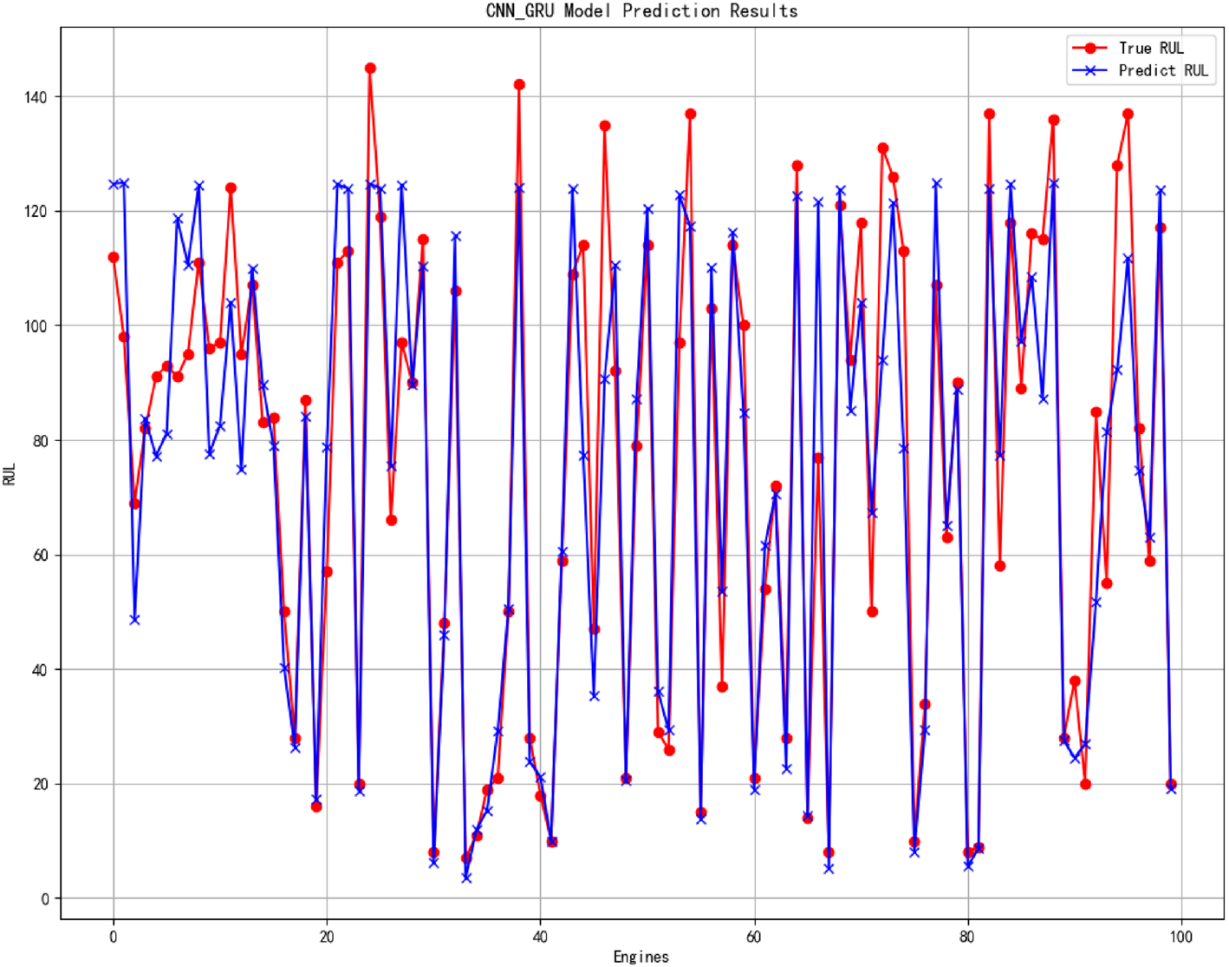
The result of RUL prediction.

The prediction results obtained by the proposed model are presented in Fig. 13. Comparative experiments were also conducted on other state-of-the-art models. The experimental findings demonstrate that, while preserving the structure of the CNN-GRU model, a combination of fine-tuning techniques is applied to parameters such as learning rate (lr), Dropout ratios (drop_CNN and drop_dense) for both CNN and fully connected layers, as well as convolution kernel size (kernel_size). This approach ensures that the evaluation metrics of the model exhibit minimal fluctuations within a narrow range: RMSE fluctuates within 5%, ${{R}^{2}}$ within 6.5%, and S_Score within 5%. The prediction outcomes are illustrated in Figs. 14 and 15.

In the presented prediction result figures, it is evident that the 1D CNN-GRU model employed in this study has accurately predicted the Remaining Useful Life (RUL) values. Notably, the prediction accuracy is higher for smaller RUL values due to intensified degradation features as the engine unit approaches its end of service life. The proposed network effectively captures these features, leading to improved prediction performance. Despite a certain margin of error, overall, the model exhibits a high level of prediction accuracy, particularly when approaching failure. This heightened predictive capability holds significant implications for enhancing operational reliability and safety, reducing maintenance costs, and optimizing system performance. Particularly in modern industrial production environments where equipment reliability and safety are paramount concerns, precise fault diagnosis, and remaining life estimation can effectively mitigate risks and minimize losses.

## Discussion

In response to the scarcity of available historical lifetime cycle samples for high-reliability equipment such as spacecraft, few-shot learning aims to construct models with excellent generalization performance in order to enable fast and generalized predictions using only a limited amount of historical lifetime cycle data. This approach overcomes the limitation of deep learning models that rely on large amounts of training data. To further enhance the predictive performance of RUL in the context of few-shot, this paper proposed a 1D CNN-GRU model directly applied to small-scale datasets for RUL prediction. Specifically, we first standardize and select monitoring parameters; then, we input the processed dataset into a constructed CNN for local feature extraction; finally, we input the extracted feature vectors into the GRU network and estimate RUL through a prediction network established by GRU. The method has the following advantages:

- We use feature selection and maximum–minimum normalization preprocessing methods to construct a feature set that includes key sensor monitoring data and input it into the CNN to avoid the computational complexity and model performance problems caused by redundant features.
- Utilizing the advantages of CNN network in the data mining field, we can extract the potential connections between non-continuous data in high-dimensional space, and avoid the drawbacks brought by the need for expertise in traditional algorithms. This method can better utilize the limited data for learning and generalization under the few-shot conditions. By combining GRU with CNN, not only can the spatial-temporal expression ability of features be enhanced, but the dynamic changes in the engine operation process can also be effectively handled.
- The GRU recurrent network model can fully consider the temporal relationship between degradation features and has a good fitting ability for regression in temporal sequence. At the same time, it is simpler and has fewer parameters than the LSTM structure, making it more suitable for RUL prediction and modeling in the context of few-shot.
- Our work has introduced a feature selection method for sensor monitoring parameters based on data distribution, correlation, and monotonicity indicators, which effectively increased the model’s focus on important features and further enhanced the prediction accuracy and robustness in the condition of small sample size.

In this paper, we conducted experimental verification using the FD001 subset of the C-MAPSS dataset and compared it with other common prediction methods. The experimental results show that in the case of fewer training samples, the 1D CNN-GRU method can achieve higher accuracy in RUL prediction and has better performance.

However, in the case of few-shot conditions, while the CNN-GRU hybrid deep neural network model can partially address issues related to insufficient data, overfitting risk, and inadequate feature mining, there are still practical limitations. Firstly, the model’s generalization ability may be inadequate, particularly when encountering unfamiliar data patterns or changes, leading to potential degradation in prediction performance. This necessitates careful evaluation of its suitability and corresponding adjustments when applying the model to new fields or environments. Secondly, incomplete representation of engine states and environmental variations under diverse operating conditions in small sample data can impact the model’s predictive capability for specific operational scenarios. Therefore, collecting and labeling more comprehensive and diverse training data is essential to enhance the model’s understanding and prediction accuracy across various operating conditions and environmental changes. Furthermore, parameter optimization and structural design become more challenging with a small sample size; thus requiring meticulous optimization tailored to different data distributions and real-world problem scenarios based on a profound comprehension of algorithm principles and mathematical foundations. Finally, limited computing resources in practical applications such as embedded systems or real-time monitoring scenarios may affect the computational efficiency and applicability scope of the hybrid deep neural network model. To strike a balance between computational efficiency and accuracy trade-off considerations could include adopting lightweight network architectures, pruning techniques,and hardware acceleration.

When addressing the limitations of hybrid deep neural network models in the context of limited sample size in the future, we plan to conduct the following research initiatives and directions. Firstly, to address the issue of insufficient model generalization, we plan to explore the introduction of cross-domain data transfer learning and adaptive learning methods to enhance the model’s suitability for different data patterns and environmental changes. Secondly, we aim to combine residual self-attention mechanisms and consider the importance changes of different attributes related to engine degradation information, assigning higher weights to critical degradation indicators to enhance the model’s understanding of operating conditions, thereby improving the accuracy of engine remaining life prediction. Meanwhile, when addressing the challenges of hyperparameter optimization and structure design, we plan to combine federated learning and deep reinforcement learning techniques to improve model performance and reduce overfitting risks. Through the exploration of these research initiatives and directions, we expect to effectively address the practical limitations of hybrid deep neural network models in limited sample size conditions and enhance the model’s performance and applicability.

## Data Availability

Data derived from asource in the publicdomain.The data underlying this article are available in https://data.nasa.gov/Aerospace/CMAPSS-Jet-Engine-Simulated-Data/ff5v-kuh6.
